# Vertical Nystagmus in the Bow and Lean Test may Indicate Hidden Posterior Semicircular Canal Benign Paroxysmal Positional Vertigo: Hypothesis of the Location of Otoconia

**DOI:** 10.1038/s41598-020-63630-3

**Published:** 2020-04-16

**Authors:** Oak-Sung Choo, Hantai Kim, Jeong Hun Jang, Hun Yi Park, Yun-Hoon Choung

**Affiliations:** 1 Department of Otolaryngology, Ajou University School of Medicine, Suwon, Republic of Korea; 2 Department of Medical Sciences, Ajou University Graduate School of Medicine, Suwon, Republic of Korea; 3 BK21 Plus Research Center for Biomedical Sciences, Ajou University Graduate School of Medicine, Suwon, Republic of Korea

**Keywords:** Neuroscience, Peripheral nervous system

## Abstract

The ‘Bow and Lean Test’ (BLT) was developed for proper diagnosis of horizontal semicircular canal benign paroxysmal positional vertigo (HSC-BPPV). Occasionally, down- and/or up-beating vertical nystagmus is observed during the BLT. This study analyzed patients who exhibited vertical nystagmus in the BLT to comprehend the clinical significance of this sign. Of 2872 patients with vertigo between 2010 and 2015, 225 patients who showed vertical nystagmus in the BLT were enrolled. All patterns of vertical nystagmus were described based on their types of BPPV. After performing therapeutic maneuvers for BPPV, remnant symptoms in the BLT findings were investigated. Of the 225 patients with vertical nystagmus, 163 were posterior semicircular canal BPPV (PSC-BPPV). Down-beating in the bowing position and no nystagmus in the leaning position (‘Down/–’) was the most common type (190 of 225 patients). In addition, the nystagmus occurred in the form of ‘–/Up’, ‘Down/Up’, and ‘–/Down’. The pattern of vertical nystagmus may be related to the position of otoconia in the canals. The location of the otoconia enables the diagnosis of hidden PSC-BPPV. Even after treatment for BPPV, patients with vertical nystagmus in the BLT tended to complain remnant vertigo symptoms (44.8% vs. 23.9%, *P* = 0.022, in PSC-BPPV; 70.0% vs. 24.0%, *P* = 0.020, in HSC-BPPV). We thought that they actually had hidden PSC-BPPV and the otoconial debris may still in the PSC; this untreated PSC-BPPV might cause the remnant symptoms. In conclusion, vertical nystagmus in the BLT may indicate the presence of PSC-BPPV. Moreover, vertical nystagmus during the BLT may occur in patients with hidden PSC-BPPV who complain of remnant vertigo symptoms. Vertical nystagmus shown in the BLT may not include the possibility of central vertigo.

## Introduction

Since the Bow and Lean Test (BLT) was first introduced^1^, it has been used to determine the affected ear in patients with horizontal semicircular canal benign paroxysmal positional vertigo (HSC-BPPV). Briefly, the BLT is based on the direction of nystagmus when a patient’s head bows and leans in a sitting position. In canalolithiasis, the affected ear is in the same direction as the nystagmus when bowing; in cupulolithiasis, the direction of nystagmus leads to the affected ear at the leaning position^1^. The BLT provides a much simpler and more precise method to identify the affected ear than confirmation of the lesion side through the comparison of nystagmus intensity in the conventional head roll test. By determining the affected ear more accurately, the BLT could ultimately improve the final remission rate of HSC-BPPV. In a follow-up study, patients who underwent both the BLT and head roll test demonstrated a final remission rate of 83.1%; however, the remission rate was 67.4% in patients diagnosed with HSC-BPPV using the head roll test alone^2^.

Notably, when the BLT was performed to diagnose the affected ear in patients with HSC-BPPV, up- or down-beating vertical nystagmus was observed in some patients. In fact, vertical nystagmus itself could be observed in the head hanging position, such as the Dix-Hallpike test, for the diagnosis of posterior semicircular canal BPPV (PSC-BPPV). Up-beating nystagmus in the Dix-Hallpike test suggests PSC-BPPV, whereas down-beating nystagmus is generally considered indicative of central nervous system abnormalities including cerebellar disorders or for the relatively rare (3% of BPPV) anterior semicircular canal BPPV (ASC-BPPV)^3–5^. Down-beating nystagmus from central lesions such as cerebellar disorder may be easily recognized from BPPV, having different characteristics of no latency, long duration, no fatigue, and no fixation. However, the clinical significance of vertical nystagmus in the BLT has not been clarified.

Therefore, this study was designed to interpret vertical nystagmus in the BLT and analyze its clinical significance. Furthermore, we evaluated the usefulness of the BLT for showing vertical nystagmus in PSC-BPPV, especially in terms of the localization of otoconia.

## Results

### Vertical nystagmus during the BLT in all enrolled patients

Table 1 shows the patterns of vertical nystagmus in all 225 patients during the first BLT. Down-beating in the bowing position and no nystagmus in the leaning position was the most common type Table 1: 190 of the total 225 patients (84.4%), 137 of the 163 PSC-BPPV patients (84.0%), and 32 of the 35 HSC-BPPV patients (91.4%). Of the 32 HSC-BPPV patients, 22 were canalolithiasis and 10 were cupulolithiasis. The nystagmus in the BLT had a short latency of 1-2 seconds, which was similar in all cases. The nystagmus with the BLT had a short latency of 1-2 seconds, which was similar in all cases. The nystagmus lasted up to 20 seconds when BPPV was diagnosed, whereas the duration sometimes exceeded 1 minute in non-BPPV patients.

**Table 1:** Direction of vertical nystagmus in BLT among all patients (N = 225).

Bowing nystagmus	Leaning nystagmus	PSC-BPPV	HSC-BPPV	ASC-BPPV	PSC- & HSC- BPPV	Non-BPPV	Total
Down	Up	5	1	0	0	0	6
Down	Down	0	0	0	0	3	3
Down	(−)	137	32	1	8	12	190
Up	Up	1	0	0	0	0	1
Up	Down	0	0	0	0	0	0
Up	(−)	0	1	0	0	2	3
(−)	Up	18	1	0	0	0	19
(−)	Down	2	0	1	0	0	3
Total	163	35	2	8	17	225	

BLT, the Bow and Lean Test; PSC-BPPV, posterior semicircular canal benign paroxysmal positional vertigo; HSC-BPPV, horizontal semicircular canal benign paroxysmal positional vertigo; ASC-BPPV, anterior semicircular canal benign paroxysmal positional vertigo; BPPV, benign paroxysmal positional vertigo.

Canalith repositioning procedures, such as the Epley maneuver, Barbeque maneuver with or without vibration, or reverse Epley maneuver, were the treatment for BPPV. The number of the repositioning maneuver required for complete remission in the groups are shown in Fig. 2a. There were no differences in the number for remission. However, there was a difference between the two groups in subjective residual symptoms. In Group B, who showed vertical nystagmus in the BLT after remission, more patients complained of remnant symptoms, though they did not continue to exhibit nystagmus in the Dix-Hallpike test (Fig. 2b). Of the patients who showed vertical nystagmus in the BLT, 35 had HSC-BPPV; 25 (72.7%) were in Group A (no nystagmus in the BLT after complete remission), whereas the remaining 10 (27.3%) were in Group B (residual vertical nystagmus in the BLT) (Fig. 3). There was also no difference in the number of the repositioning procedures required for the remission of HSC-BPPV (Fig. 3a). Group B HSC-BPPV patients were more likely to complain of remnant dizziness after successful otolith reduction (Fig. 3b).

### Vertical nystagmus in the BLT in non-BPPV patients

Among 1848 non-BPPV patients, vertical nystagmus in the BLT was observed in 17 patients (0.9%) (Table 1). Of those 17 patients, three (17.6%) were men and 14 (82.4%) were women. Four patients (23.5%) had a previous history of one or more occurrences of BPPV, and five (29.4%) were using sedative medications for the treatment of other disorders.

### Radiologic evaluations for differential diagnosis of central origin vertigo

Brain imaging, such as CT or MRI, was performed in 113 of 225 patients. Ninety-six of the 208 patients diagnosed with BPPV underwent brain evaluation by CT or MRI. Sixty-one (63.5%) of the 96 BPPV patients showed normal findings in this evaluation; other findings were the following: senile changes such as mild diffuse atrophy or vessel stenosis in 18 (18.8%), acute infarction in 1 (1.0%), brain tumor in 2 (2.1%), and traumatic brain hemorrhage in 12 (12.5%) patients.

Of the 96 BPPV patients, 78 were diagnosed with PSC-BPPV. Of these 78 patients, most had favorable progress after treatment of PSC-BPPV. Only four patients (two had traumatic brain hemorrhage, one had mild vessel stenosis, and one had diffuse brain atrophy) complained of residual dizziness, although none had persistent vertical nystagmus. Fifteen patients were diagnosed with HSC-BPPV. Twelve patients had normal findings; the remaining three had vessel stenosis, a brain tumor (meningioma), and mild diffuse atrophy. Only the 86-year-old patient with brain atrophy had continuous down-beating nystagmus in the BLT, with residual dizziness after canalith repositioning maneuvers. In the other two patients, nystagmus disappeared after treatment. Three patients had mixed type BPPV, but all three had normal brain examination findings. All 17 non-BPPV patients also underwent brain examinations, but none had abnormal findings that could cause central vertigo. Therefore, down-beating nystagmus in the BLT is less commonly related to central lesions than previously suspected.

## Discussion

### PSC-BPPV and vertical nystagmus in the BLT

**Figure 1:**
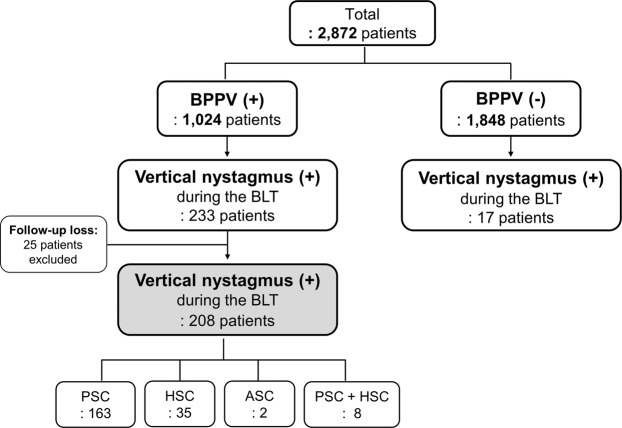
Flow chart. Patients who showed vertical nystagmus in the BLT, along with their diagnosis. Analysis in this study was performed in 208 BPPV and 17 non-BPPV patients who exhibited vertical nystagmus in the BLT. BLT, Bow and Lean Test; BPPV, benign paroxysmal positional vertigo. PSC, posterior semicircular canal; HSC, horizontal semicircular canal; ASC, anterior semicircular canal.

Since the initial proposal of the BLT in 2006^1^, we have performed this test in all patients suspected of BPPV. However, we have often encountered patients with up- or down-beating vertical nystagmus in the BLT; thus, we performed this study to investigate the mechanism and clinical application of this vertical nystagmus. Of the 1024 patients diagnosed with BPPV, vertical nystagmus in the BLT was observed in approximately 20% (Fig. 1). The most common type of vertical nystagmus was down-beating in the bowing position and none in the leaning position, “Down/–”, in the BLT. Analysis of this pattern might provide clues regarding the mechanisms of vertical nystagmus in the BLT. As shown in Fig. 4, the BLT can induce the movement of otoconia in PSC-BPPV. Otoconia in the PSC migrates toward the ampulla when the patient bows, which can cause ampullopetal flow and, in turn, produce down-beating nystagmus (Fig. 4). However, in the leaning position, it is difficult to provoke ampullopetal or ampullofugal flow sufficiently to induce nystagmus, because its movable angle is limited relative to that in the bowing position. Thus, the presence of vertical nystagmus indicates the possibility of PSC-BPPV.

However, why did this inconsistent vertical nystagmus in the BLT occur? As illustrated in Fig. 4, the site of the otoconia, which are thought to cause down-beating nystagmus in the bowing position, is the dependent position for particles in the PSC. Thus, because most PSC-BPPVs would involve this location, down-beating nystagmus should be frequently observed when performing the BLT in patients with a lesion in the PSC; however, vertical nystagmus did not occur in all patients with PSC-BPPV. To explain this, we consider the direction and influence of gravity in maneuvers used to diagnose BPPV. These diagnostic maneuvers, the Dix-Hallpike test for PSC-BPPV and the head roll test for HSC-BPPV, can change the direction of gravity acting on each semicircular canal. The PSC is completely reversed when performing the Dix-Hallpike test, and the direction of gravity is similarly changed. In the head roll test, the gravity direction is also altered when the horizontally located HSC becomes perpendicular to the ground. This change in the direction of gravity, in addition to the movement of otoconia, can create an endolymph ampullopetal or ampullofugal flow that enables the development of nystagmus. Conversely, considering the movement of PSC in the BLT, compared to the Dix-Hallpike test or head roll test, there is little change in the direction of gravity on the canal. Eventually, changes in endolymphatic flow occur dependently by the otoconia; thus, vertical nystagmus may not appear in the BLT unless the debris are of sufficient size to cause this altered flow.

By these ideas, we can propose the mechanisms by which other types of nystagmus occur in the BLT. We assume that debris should be sufficiently large or numerous to create a flow that can provoke nystagmus solely by movement of otoconia. The location of otoconia debris should be considered because it determines the aspect of endolymphatic flow. In the PSC, we suppose that otoconial masses are located at locations 1, 2, 3, and 4, as shown in Fig. 5a. In the Dix-Hallpike test, each otoconia moves in the direction of the arrow, and all four movements are similar (Fig. 5b). All four otoconia cause ampullofugal flow, resulting in up-beating nystagmus and a diagnosis of PSC-BPPV, as is well-known. Therefore, although these otoconia show slightly different latencies, all demonstrate the same up-beating nystagmus, regardless of the initial locations of otoconia (‘Nystagmus in the Dix-Hallpike test’ in Table 2).

**Table 2:** Possible nystagmus types according to the location of otolith.

Location No.	Nystagmus in the Dix-Hallpike test	Nystagmus in Bowing	Nystagmus in Leaning
1	Up-beating	No nystagmus	No nystagmus Up-beating
2	Up-beating	No nystagmus Down-beating	Up-beating No nystagmus
3	Up-beating	Down-beating	No nystagmus
4	Up-beating	No nystagmus	Down-beating

In contrast, in the bowing position, each otoconia moves in a distinct manner (Fig. 5c). The otoconia at location 1 does not move. The otoconia at location 2 also may not move or may cause some flow changes along the red arrow in Fig. 5c. Thus, the otoconia at location 1 would not show any nystagmus at the bowing position; the otoconia at location 2 may create no nystagmus or may create some ampullofugal flow resulting in down-beating nystagmus. The otoconia at location 3 would be distinctly directed to the ampulla and thus may induce down-beating nystagmus; however, the otoconia at location 4 would demonstrate no movement of debris, and nystagmus would not occur (‘Nystagmus in Bowing’ in Table 2).

Nystagmus at the leaning position can be explained with the same rationale (Fig. 5d). The first otoconia can create ampullofugal flow and cause up-beating nystagmus. However, even if the first otoconia is located very closed to the ampulla, this does not indicate PSC cupulolithiasis, because cupulolithiasis cannot present the type of movement proposed in this study due to debris attachment to the cupula of the canal^6,7^. The first otoconia should be recognized as a canalolithiasis close to the ampulla. The presence of otoconia at locations 2 and 3, which are dependent positions, may result in no movement in the leaning position. Rarely, the second otoconia might demonstrate up-beating nystagmus if it moves slightly down (red arrow in Fig. 5d). The fourth otoconia produces an ampullopetal flow that cause up-beating nystagmus (‘Nystagmus in Leaning’ in Table 2).

The most common form, “Down/–”, is possible when otoconia is present at location 2 or 3. In particular, when otoconia is present at location 2, nystagmus occurs in neither the bowing nor leaning positions. In PSC-BPPV, since most otoconia are present at location 2 or 3, the combination of “Down/–” or “–/–” occurs most frequently. In Table 1, five patients with “Down/Up” nystagmus would have otoconia at location 2, and 18 patients with “–/Up” nystagmus had PSC-BPPV at location 1. In addition, “–/Down” can be thought of as otoconia in location 4; it is unlikely that debris would be present in that location, so only two patients were included. Table 2 shows that there are no combinations of “Down/Down”, “Up/Up”, “Up/Down”, or “Up/–” nystagmus; indeed, none of the patients had “Down/Down”, “Up/Down”, or “Up/–” nystagmus in PSC-BPPV (Table 1). In this context, our hypothesis was suitable, except that only one patient exhibit “Up/Up” nystagmus. We presume that this one exceptional case was a recording error.

In summary, vertical nystagmus that occurred in the BLT was associated with PSC-BPPV. Down-beating in the bowing and no nystagmus in the leaning, Down/–, was the most common pattern of the nystagmus. Such pattern of the nystagmus seemed to depend on the location of the otoconia in the PSC.

### Atypical PSC-BPPV and vertical nystagmus in the BLT

**Figure 2:**
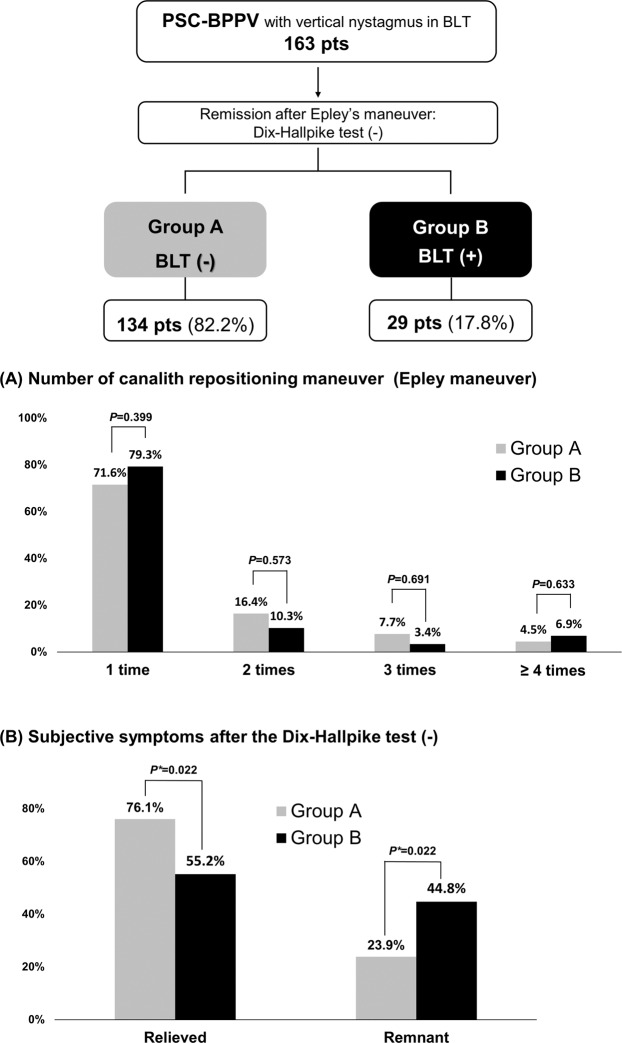
Vertical nystagmus in BLT in patients with posterior semicircular canal benign paroxysmal positional vertigo (n = 163). After treatment, remission was confirmed by the Dix-Hallpike test. In the 134 patients (82.2%, Group A), vertical nystagmus in the BLT also disappeared; however, it persisted in the remaining 29 patients (17.8%, Group B). Patients in Group B tended to complain of remnant symptoms after remission (44.8% in Group B and 23.9% in Group A, p = 0.022). BLT, Bow and Lean Test; PSC, posterior semicircular canal.

**Figure 3:**
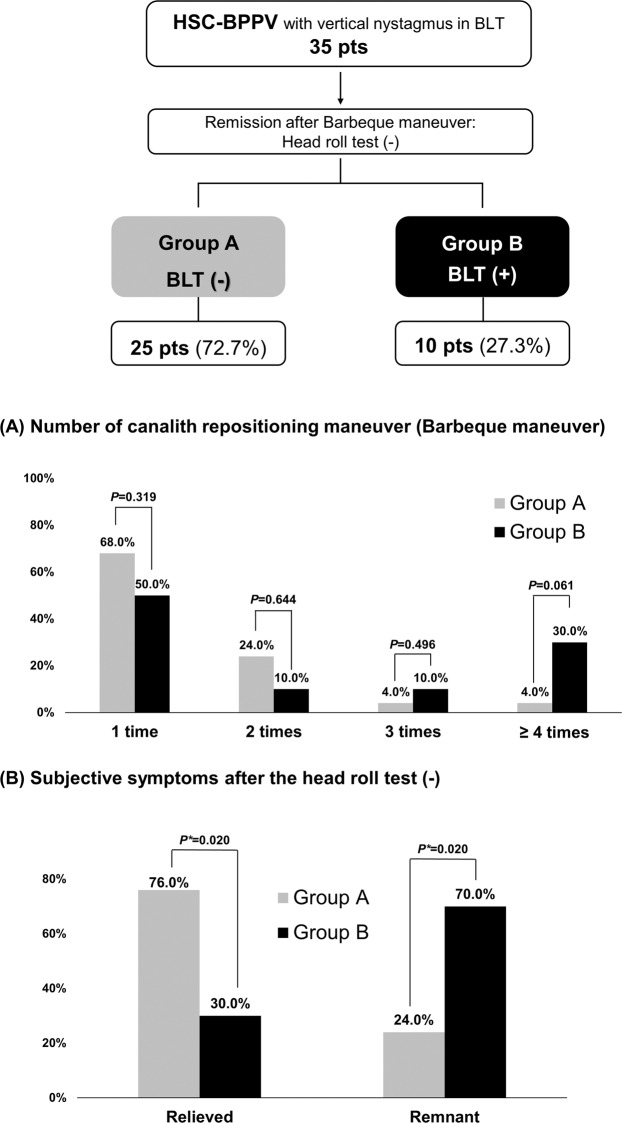
Vertical nystagmus in the BLT in patients with horizontal semicircular canal benign paroxysmal positional vertigo (n = 35). After treatment, remission was confirmed by the head roll test. In 25 patients (72.7%, Group A), vertical nystagmus did not occur in the BLT, whereas it persisted in the remaining 10 patients (27.3%, Group B). Patients in Group B were likely to complain of remnant symptoms after remission (70.0% in Group B and 24.0% in Group A, *p* = 0.020). BLT, Bow and Lean Test; HSC, horizontal semicircular canal.

After finishing treatment for BPPV, some patients still exhibited persistent down-beating nystagmus in the bowing position; these patients tended to complain of remnant symptoms (Figs. 2 and 3). Vannuchi et al. suggested a variant type of PSC-BPPV that exhibited ‘torsional vertical down-beating nystagmus’ in the Dix-Hallpike test; they defined this as apogeotropic PSC-BPPV (A-PSC-BPPV)^8^. In a follow up study, they reported the clinical features, mechanism, and treatment of A-PSC-BPPV^9^. Since then, this atypical PSC-BPPV has often been reported^10,11^. Vannuchi et al. hypothesized that the otoconial mass was trapped in a non-ampullary arm of the PSC, close to common crus where the PSC and ASC met (Fig. 5e). They suggested that the trapped otoconia could produce ‘torsional vertical down-beating nystagmus’ by ampullopetal flow during the Dix-Hallpike test^8,9^. We speculate that our patients who complain of remnant symptoms could be affected by the same principle. Earlier in the Discussion of the present paper, we suggested that a relatively large otoconia, which can produce endolymph flow, could induce vertical nystagmus in the BLT. When the otoconia are extracted through the common crus to the utricle in the Epley maneuver, some otoconial masses might be trapped at the location suggested by Vannuchi et al. (Fig. 5e). Figure 5f,g show the Dix-Hallpike test and BLT in a situation where the debris is trapped. Vannuchi et al. found that otoconia could move along the red arrow in Fig. 5f and that the ampullopetal flow could cause down-beating nystagmus in the Dix-Hallpike test^9^. However, we suspect that if the debris was trapped but could move within a limited distance, the ampullopetal flow could be produced; conversely, if otoconia were trapped within a smaller space, the debris could neither move nor produce the flow. Even the ampullopetal flow here counteracted the direction of gravity, which might further offset the magnitude of the flow. Although we agree that the debris could be trapped in the canal, there have been many patients who do not exhibit ‘torsional vertical down-beating nystagmus’ in the Dix-Hallpike test. These patients exhibited ‘hidden A-PSC-BPPV’. In these patients, because nystagmus was not observed in the Dix-Hallpike test, the practitioners judged that PSC-BPPV had been resolved despite the persistence of otoconia within the canal.

**Figure 4:**
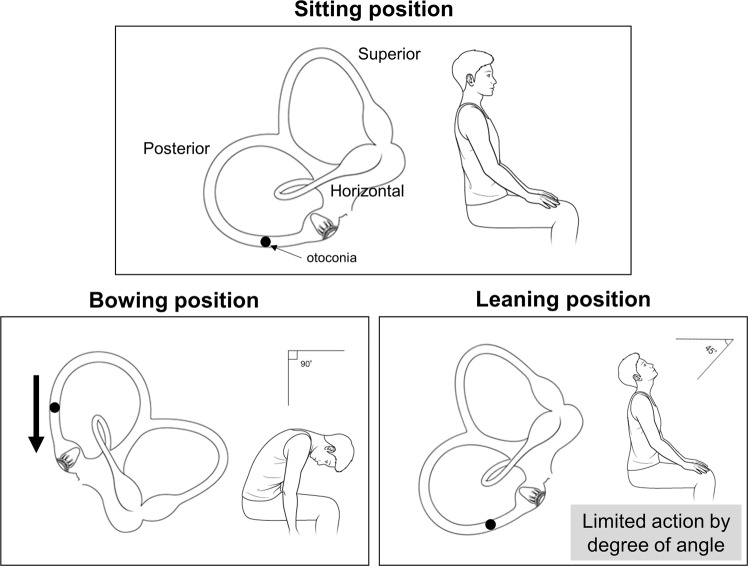
Possible mechanisms for certain directions of vertical nystagmus in the BLT in patients with benign paroxysmal positional vertigo. In the bowing position, the otoconia moves toward the ampulla, resulting in ampullopetal flow. This flow provokes down-beating nystagmus. Conversely, in the leaning position, the angles at which the head can be moved are limited, causing difficulty in movement of the otoconia located in the dependent position in the posterior semicircular canal. BLT, Bow and Lean Test.

**Figure 5:**
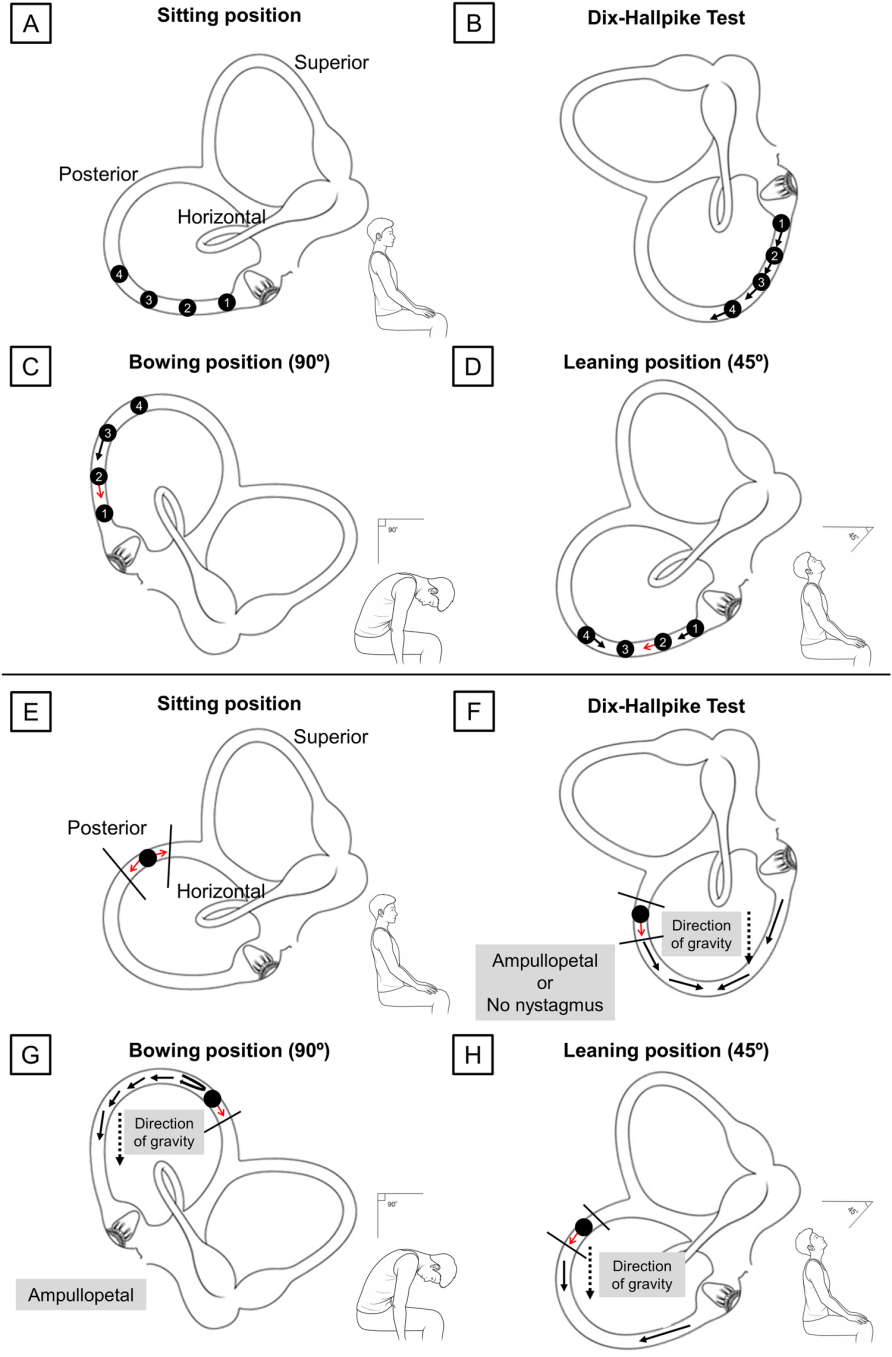
(**a–d**) Possible movement based on locations of otoconia in the Dix-Hallpike test and BLT. (**a**) Four presumed locations in which otoconia can be placed. (**b**) In the Dix-Hallpike test, all otoconia move in the direction of the arrow, causing ampullofugal flow. (**c)** In the bowing position, the otoconia in locations 1 and 4 would not move. The otoconia in location 3 moves toward the ampulla. The otoconia in location 2 might move down, resulting in ampullopetal flow; the debris may not move, depending on the degree of the bowing angle (red arrow indicates whether it can move in that direction). (**d**) In the leaning position, the otoconia in locations 1 and 4 can be moved away from and toward the ampulla, respectively. The otoconia in location 3 would remain there. The otoconia in location 2 may move, depending on the degree of the leaning angle. (**e**–**h**) Effect of trapped otoconia on endolymphatic flow in the Dix-Hallpike test and BLT. (**e**) Otoconia can be trapped in the non-ampullary distal part of the PSC. (**f**) In the Dix-Hallpike test, the trapped otoconia might create ampullopetal flow when moving downward; however, if they are trapped within a smaller space, the flow may be absent. (**g**) Normally, the endolymph should flow to the non-ampullary distal part on the PSC; however, trapped otoconia interfere with this flow, allowing a greater amount of endolymph to flow toward the ampulla in the bowing position. (**h**) In the leaning position, similar to the Dix-Hallpike test, the otoconia may migrate to induce ampullopetal flow and may exhibit minimal motion. BLT, Bow and Lean Test; PSC, posterior semicircular canal.

If the BLT is performed when otoconia are trapped, the flow (black arrows in Fig. 5g) cannot reach the common crus due to the presence of otoconia in the non-ampullary distal portion of the PSC. When the flow eventually returns and the effect of gravity adds to the returning flow, the ampullopetal flow will be stronger, which creates down-beating bowing nystagmus (Fig. 5g). However, it is somewhat difficult to predict the direction of the flow induced in the leaning position. Flow can occur along the direction of arrows in Fig. 5h. As the otoconia gradually recedes farther from the ampulla, it becomes difficult to make a flow that can stimulate the ampulla in the leaning position, If the otoconia exit from the non-ampullary PSC in the leaning position, they can cause ampullopetal flow, resulting in down-beating nystagmus. Indeed, one patient showed this down-beating leaning nystagmus, whereas the remaining 28 patients demonstrated no nystagmus in the leaning position (Group B in Fig. 2). In these patients, the otoconial debris might persist in the canal; they were thus more likely to complain of remnant symptoms.

### HSC-BPPV and vertical nystagmus in the BLT

We speculate that the 32 down-beating nystagmus patients with HSC-BPPV actually exhibited multi-canal involvement BPPV with hidden A-PSC-BPPV or weak PSC-BPPV. Because the A-PSC-BPPV was hidden, these patients were diagnosed with HSC-BPPV alone. Regarding the possibility of weak PSC-BPPV, when performing the Dix-Hallpike test in PSC-BPPV, vertical nystagmus may occasionally not occur, and torsional beating alone may appear^12^. Furthermore, if HSC- and PSC-BPPV are concomitantly present, HSC can be stimulated simultaneously in the Dix-Hallpike test^13^. In particular, if HSC-BPPV cupulolithiasis is combined, the nystagmus caused by cupulolithiasis can offset the torsional nystagmus of PSC-BPPV and weaken the beating in the Dix-Hallpike test, causing the test results to be negative. Therefore, although otoconial masses were concurrently involved in HSC and PSC, patients with presumable hidden or weak PSC-BPPV may not exhibit positive results in the Dix-Hallpike test; if the multi-canal involvement of PSC- and HSC-BPPV is clearly identified, “Down/–” may appear in the BLT. As we expected, this form occurred in eight patients with mixed PSC- and HSC-BPPV (Table 1). Furthermore, of the 35 patients, we recognized that two had been transposed from HSC- to PSC-BPPV, and one was later diagnosed with multi-canal involvement; however, these three might also have initially exhibited PSC-BPPV.

BPPV could be spontaneously resolved without the canalith repositioning procedures^14^. In the 35 patients in Fig. 3, after treatment of HSC-BPPV alone, vertical nystagmus was not present in the BLT in 72.7% of these patients. Those who exhibited PSC-BPPV may have undergone spontaneous resolution. However, some patients exhibited persistent nystagmus due to weak or hidden forms of PSC-BPPV; this may have been the cause of complaints of remnant symptoms in Group B (Fig. 3). The hypothesis that hidden A-PSC-BPPV or weak PSC-BPPV may cause down-beating bowing nystagmus is also applicable to the 17 non-BPPV patients who showed vertical nystagmus. Of these, 15 showed down-beating nystagmus, which was the same pattern exhibited in patients with the weak or hidden PSC-BPPV. This explanation is further supported by the observation that all 17 patients did not have a central lesion and that five of these patients had a history of BPPV.

## Summary

The BLT not only determines the affected side in HSC-BPPV but may be also useful for detecting PSC-BPPV. Vertical nystagmus in the test, especially down-beating nystagmus during the bowing position, may indicate the presence of PSC-BPPV. Moreover, vertical nystagmus during the BLT may occur in patients with hidden PSC-BPPV who complain of remnant vertigo symptoms. Vertical nystagmus shown in the BLT may not always indicate the possibility of central vertigo.

## Methods

### Patients

Among 2872 patients with vertigo who visited the Dizziness Clinic in the Department of Otolaryngology, Ajou University Hospital (Suwon, Republic of Korea), between August 2010 and July 2015, 1024 were diagnosed with BPPV, and 1848 were not (non-BPPV). Patients who showed vertical nystagmus in the BLT on the first day of their hospital visit, regardless of the final diagnosis, were included in this study. Two hundred and eight (20.3%) of the 1024 BPPV patients were included in the BPPV group, and 17 (0.9%) of the 1848 non-BPPV patients were included in the non-BPPV group (Fig. 1). The medical records of the 225 patients were retrospectively reviewed. The patients’ basic demographics and direction of nystagmus in the diagnostic maneuvers were analyzed. Either brain computed tomography (CT) or brain magnetic resonance imaging (MRI) of 113 patients were included. Final treatment outcomes were also included in this analysis. This study was approved by and performed according to the guideline of the Institutional Review Board of Ajou University Hospital. And, the Institutional Review Board of Ajou University Hospital also waived the informed consent for the study. The approval code issued by the Institutional Review Board of Ajou University Hospital is AJIRB-MED-MDB-19-221.

### Diagnosis and treatment protocol of BPPV

The diagnostic maneuvers—Dix-Hallpike test, head roll test, and the BLT—were performed on the first day of each patient’s hospital visit by members of our Dizziness Clinic who had more than 10 years of clinical experience in evaluation of patients with vertigo. Nystagmus was tracked and recorded via electronystagmography (Micromedical Technologies, Inc., Chatham, IL, USA) using a computerized video eye-movement recorder (SLMED, Seoul, Republic of Korea). PSC-BPPV was diagnosed, where nystagmus beat vertically upward and torsionally downward in the Dix-Hallpike test. When the torsional movement was counterclockwise during the right side test, the right ear was affected; clockwise torsional movement beating during the left side test indicated that the left ear was affected^3,4,12^. Nystagmus that was down-beating and torsionally counterclockwise during the left side test suggested ASC-BPPV involving the right ear, nystagmus that was down-beating and torsionally clockwise during the right side test suggested ASC-BPPV involving the left ear^12^.

HSC-BPPV was first confirmed by geotropic or apogeotropic paroxysmal nystagmus provoked by the head roll test when the head was turned from the supine to both lateral positions. Geotropic movement of the eyeball was indicative of canalolithiasis, whereas apogeotropic movement suggested cupulolithiasis. The affected ear was determined by using the BLT protocol introduced by Choung *et al*.^1^. The BLT evaluated the direction of nystagmus when patients bowed their heads over 90° to 120° (bowing nystagmus) and leaned their heads backward over 45° to 60° (leaning nystagmus) in the sitting position. For canalolithiasis, the affected ear was determined to be in the same direction as the bowing nystagmus and in the direction opposite to the leaning nystagmus. In patients who did not show bowing or leaning nystagmus, or patients who showed vertical nystagmus, the affected ear was the side with more intense nystagmus in canalolithiasis and with less intense nystagmus in cupulolithiasis. Regardless of the results of the Dix-Hallpike test and head roll test, the BLT was performed in all 2872 patients. Notably, the BLT was the first maneuver performed, because the Dix-Hallpike test and head roll test could cause changes in otoconial location.

Patients with BPPV were treated with the canalith repositioning procedures based on the location of otoconia; the Epley maneuver, Barbeque maneuver, Barbeque maneuver with vibration, and reverse Epley maneuver were applied to patients with PSC-BPPV, canalolithiasis of HSC-BPPV, cupulolithiasis of HSC-BPPV, and ASC-BPPV, respectively. Non-BPPV patient were individually managed based on the possible causes of vertigo.

### Analyses and statistical methods

In the 225 patients included in this study, all aspects of vertical nystagmus in the BLT were classified in accordance with each patient’s diagnosis (Table 1). The findings of the 117 patients who underwent brain CT or MRI were also recorded. Patients who vertical nystagmus in the BLT with PSC-BPPV (163 patients) and HSC-BPPV (35 patients) were further analyzed. When complete remission was achieved after one or more the repositioning maneuver, the BLT was performed again, and the presence of vertical nystagmus was investigated. Group A comprised patients who had no nystagmus in any of the tests (Dix-Hallpike test, head roll test, or the BLT). Group B comprised patients who had no nystagmus in the Dix-Hallpike test or head roll test but had vertical nystagmus in the BLT (Figs. 2 and 3). Chi-square analysis was used to compare between Group A and B.

All statistical analyses were performed using IBM SPSS Statistics for Windows (version 21.0, Armonk, NY, USA), and p values less than 0.05 were considered statistically significant.
